# On the Cure of the Febris Intermittens, by the Ammonia Preparata

**Published:** 1806-04

**Authors:** Philip Rainbird

**Affiliations:** Cawston, Norfolk


### On the Cure of the Febris Intermittens, by the Ammonia Preparata; *communicated by* Mr. Philip Rainbird, *of Cawston, Norfolk.*

P. N. ætat. 30, of rather small stature, thin habit, and dark complexion, was attacked with the Ague while employed in the Fens of Lincolnshire last autumn. Being unable to continue his occupation, he came to this place greatly reduced, and emaciated from the violence of the disease, which had been upon him three or four months; and he had not taken, as he said, any thing for it. When he applied to me, in the latter part of October, the intermittent had assumed the tertian type; and to the symptoms, which characterize ague in general, were added the unfavourable circumstances of a hard tumid abdomen, rather painful to the touch; great listlessness; frequent nausea; and total loss of appetite. Suspecting, from the state of the body and the appearance of the countenance, that the liver was unsound, or obstructed, I concluded the administering of the bark to be inadmissible; and having observed, about a year ago, the quick and unexspected recovery of a boy, ætat. 12, from a quartan ague, to whom the ammonia and the pulv. cort. peruv. were given in equal parts (only five grains of each) three or four times in the day—my attention was excited to a consideration of the efficiency of the ammoniacal carbonat, in that case; I therefore, in this, resolved to make trial of it alone in this form: ℞. Ammon. præp. ʒij. cons. cynosbat. q. s. ut fiat massa in pilulas xxiv. dividend. Two of these pills were taken quater in die quotidie, without any regard to the different stages of the intermittent. Six days had not elapsed, before my patient was much better in every respect. But complaining of a scalding of his water, which no doubt arose from the causticity of the alkali, during the rest of the time he took it, I omitted the the conserve in the above formula, and substituted two drachms of the pulv. gummi arabici, and made it into the same number of pills, with q. s. syrupi simplicis, which entirely obviated the affection. The man continued the medicine fourteen days longer, when every symptom of ague disappeared, and he now remains in better health than he had for some time been before his ague.

It would be highly absurd to form a positive conclusion in favour of the ammonia from the instance of a single case; but as my patient, being a poor man, had not the advantage of those powerful auxiliaries, wine, and a nourishing diet, usually combined where the bark is administered, I think the cure, as effected solely by the alkali, has a *great claim* on medical attention.

The ague being very rare where I am situated, my experience must be slow; I have therefore submitted the matter to the consideration of those whose situations may give them ample opportunity speedily to bring it to a decision. At all events, I intend to proceed in the probation of the ammonia; and, with your permission, through the medium of this Journal, at a future period, I shall offer some further observations on the subject.

*Feb.* 22. 1806.

